# A comparative study of the far lateral approach of uni-portal non-coaxial spinal endoscopic surgery versus percutaneous endoscopic transforaminal discectomy (PETD) for L5/S1 foraminal stenosis with high iliac crest: a retrospective cohort study

**DOI:** 10.1007/s00264-026-06840-0

**Published:** 2026-05-19

**Authors:** Jie Zhang, Xuanwen Liu, En Song, Dan Chen, Hongda Zhou, Qin Luo

**Affiliations:** 1 Department of Orthopaedics, Chengdu 363 Hospital, Chengdu, China; 2 Department of Sports Medicine, First Affiliated Hospital of Kunming Medical University, Kunming, China

**Keywords:** Uni-portal non-coaxial spinal endoscopic surgery, Far lateral approach, Transforaminal approach, High iliac crest, L5/S1, Foraminal stenosis

## Abstract

**Objective:**

To compare the clinical efficacy and radiological outcomes of the far lateral approach of uni-portal non-coaxial spinal endoscopic surgery versus traditional percutaneous endoscopic transforaminal discectomy (PETD) for the treatment of L5/S1 foraminal stenosis with a high iliac crest.

**Methods:**

A retrospective analysis was conducted on 82 patients with L5/S1 foraminal stenosis and a high iliac crest treated at our hospital from June 2023 to June 2025. Patients were divided into a uni-portal non-coaxial spinal endoscopic surgery group (*n* = 42) and a PETD group (*n* = 40) based on the surgical procedure. Operative time, the fluoroscopy time estimated blood loss, length of hospital stay, and complication rates were recorded and compared between the two groups. Clinical outcomes were evaluated using the Visual Analogue Scale (VAS) for leg pain, the Oswestry Disability Index (ODI), and the modified MacNab criteria. Radiological assessments included postoperative foraminal area, facet joint preservation rate, and segmental stability.

**Results:**

All patients were followed up for at least 12 months. The uni-portal non-coaxial spinal endoscopic surgery group had a significantly shorter operative time (68.5 ± 12.3 min vs. 92.6 ± 18.4 min, *P* < 0.001) and significantly fewer fluoroscopy time (5.4 ± 1.5 vs. 15.8 ± 4.2, P < 0.001) compared to the PETD group. There were no significant differences between the two groups in estimated blood loss (42.5 ± 15.3 mL vs. 40.2 ± 16.1 mL, P 0.05) or length of hospital stay (3.2 ± 1.1 days vs. 3.6 ± 1.4 days, P 0.05). Both groups showed significant improvement in VAS and ODI scores at all postoperative time points compared to preoperative values (*P* < 0.05). At one week postoperatively, the uni-portal non-coaxial spinal endoscopic surgery group had noticeably better leg pain VAS scores than the PETD group (2.2 ± 0.7 vs. 3.3 ± 1.3, *P* < 0.01), while clinical outcomes were comparable between the two groups at three, six and 12 months postoperatively (P 0.05). The excellent-to-good rate according to the modified MacNab criteria was 90.5% in the uni-portal non-coaxial spinal endoscopic surgery group and 87.5% in the PETD group (P 0.05). Radiologically, the uni-portal non-coaxial spinal endoscopic surgery group demonstrated a noticeably larger postoperative foraminal area (79.8 ± 13.2 mm^2^ vs. 63.5 ± 12.1 mm^2^, P < 0.001) and a noticeably higher facet joint preservation rate (93.5% vs. 75.8%, P < 0.01) compared to the PETD group. The complication rate was 7.1% in the uni-portal non-coaxial spinal endoscopic surgery group and 17.5% in the PETD group (P 0.05).

**Conclusion:**

Both the far lateral approach of uni-portal non-coaxial spinal endoscopic surgery and PETD are effective treatments for L5/S1 foraminal stenosis with a high iliac crest, yielding satisfactory clinical outcomes. Compared to PETD, uni-portal non-coaxial spinal endoscopic surgery offers advantages including shorter operative time, less fluoroscopy, more thorough foraminal decompression, and better preservation of the facet joint, making it a valuable and comparable alternative with additional perioperative benefits for managing pathologies in the L5/S1 region with a high iliac crest.

## Introduction

Lumbar foraminal stenosis (LFS) is a common cause of low back and leg pain, accounting for 8%−25% of surgeries for degenerative lumbar spine conditions [1–3]. The L5/S1 segment, due to its specific anatomical location, is a common site for foraminal stenosis [4]. The L5 nerve root traverses the L5/S1 foramen; when compressed due to disc herniation, ligamentum flavum hypertrophy, or facet joint hypertrophy and infolding, it can lead to radiating pain, paraesthesia, and motor weakness in the L5 nerve root distribution [5, 6].

Surgical treatment of the L5/S1 segment faces unique anatomical challenges, including a high iliac crest, transverse process hypertrophy, foraminal stenosis, and anatomical variations of the L5 nerve root [7, 8]. Among these, a high iliac crest is the most common obstacle for the transforaminal approach. Traditionally, a high iliac crest is defined when the iliac crest apex is above the level of the middle one-third of the L5 vertebral body, which can hinder the safe placement of percutaneous instruments, increasing surgical difficulty and the risk of nerve injury [9, 10].

Percutaneous endoscopic transforaminal discectomy (PETD), which emerged in the 1990 s, has become a mainstream minimally invasive technique for treating lumbar disc herniation and foraminal stenosis [11, 12]. However, for patients with a high iliac crest at L5/S1, PETD often faces challenges such as iliac bone obstruction preventing ideal working cannula placement, necessitating extensive foraminoplasty or even facetectomy, which increases the risk of postoperative segmental instability [13, 14]. Studies have shown that for patients with a high iliac crest, the traditional transforaminal approach requires a more complex puncture trajectory and more fluoroscopy, significantly increasing surgical difficulty.

To overcome this anatomical obstacle, various modified approaches have been explored, including the interlaminar approach, contralateral translaminar approach, and 45° tilted transforaminal approach [15–18]. Specifically, the 45° tilted transforaminal approach (45°TELD) is designed for L5/S1 with a high iliac crest, with the puncture point located 8–10 cm lateral to the posterior midline at a 45° angle to the coronal plane, effectively avoiding iliac crest obstruction, shortening the puncture path, and reducing fluoroscopy.

Uni-portal non-coaxial spinal endoscopic surgery, also known as arthroscopic-assisted uni-portal spinal surgery (AUSS), is a rapidly developing spinal endoscopic technique. This technique utilizes the Wiltse approach to directly reach the extraforaminal region, effectively bypassing the high iliac crest and allowing for direct decompression of the L5 nerve root [19, 20]. Unlike traditional coaxial endoscopes, UNSES/AUSS adopts a non-coaxial design, with the operation channel and the observation channel separated. The endoscope and the operation instruments enter the same working channel respectively, providing a larger operation space and a more flexible surgical perspective.This technique is particularly suitable for revision surgeries, complex reconstructions (e.g., annulus fibrosus repair, dural repair), combining the visual field advantages of biportal endoscopy with the minimally invasive nature of uniportal endoscopy.

Currently, there are few comparative studies on the far lateral approach of uni-portal non-coaxial spinal endoscopic surgery versus PETD for treating L5/S1 foraminal stenosis with a high iliac crest. This study retrospectively analyzes the clinical data of 82 patients to compare the clinical efficacy, radiological outcomes, and complications of the two techniques, aiming to provide evidence-based guidance for surgical approach selection in L5/S1 foraminal stenosis with a high iliac crest.

## Materials and methods

### General data

Data from patients who underwent endoscopic L5/S1 foraminal decompression at our hospital between June 2023 and June 2025 were retrospectively reviewed.

**Inclusion criteria:** (1) Unilateral L5 nerve root compression symptoms, including radiating pain, paraesthesia in the anterolateral thigh, medial leg, and medial dorsum of the foot, or weakness of the extensor hallucis longus; (2) Imaging confirmation of L5/S1 foraminal stenosis (foraminal height < 15 mm or foraminal area < 75 mm^2^) [21], and presence of a high iliac crest (iliac crest apex above the level of the middle one-third of the L5 vertebral body); (3) Failure of conservative treatment for at least six weeks; (4) Follow-up duration ≥ 12 months.

**Exclusion criteria:** (1) Multilevel foraminal stenosis requiring simultaneous surgery; (2) Grade II or higher lumbar spondylolisthesis; (3) Previous surgery at the same level; (4) Lumbar infection, tumour, or fracture; (5) Severe osteoporosis (T-score < −2.5); (6) Psychiatric disorders or inability to comply with follow-up.

A total of 82 patients were included and divided into two groups based on the surgical procedure: the uni-portal non-coaxial spinal endoscopic surgery group (*n* = 42) and the PETD group (*n* = 40). There were no statistically significant differences in preoperative general data between the two groups (P 0.05), indicating comparability (Table 1). This study was approved by the hospital's ethics committee, and all patients provided written informed consent before surgery.

**Table 1:** Comparison of preoperative general data between the two groups

Parameter	UNSES/AUSS Group (*n* = 42)	PETD Group (*n* = 40)	t/χ^2^ value	*P* value
Age (years, mean ± SD)	58.6 ± 10.2	58.6 ± 10.2	0.294	0.770
Sex (Male/Female)	24/18	22/18	0.035	0.851
BMI (kg/m^2^, mean ± SD)	24.8 ± 3.1	25.2 ± 3.4	−0.562	0.576
Disease Duration (months, mean ± SD)	8.5 ± 4.2	9.1 ± 5.0	−0.591	0.557
Affected Side (Left/Right)	23/19	21/19	0.031	0.860
Leg Pain VAS (score, mean ± SD)	6.8 ± 1.2	6.9 ± 1.3	−0.363	0.718
ODI (%, mean ± SD)	58.5 ± 10.6	59.2 ± 11.2	−0.294	0.770
High Iliac Crest Type (I/II/III)	18/16/8	17/15/8	0.012	0.994

The assignment to the UNSES/AUSS or PETD group was not randomized but was based on a combination of factors including the surgeon’s assessment of the individual patient’s anatomy (e.g., extreme iliac crest height, facet morphology), the prevailing surgical protocol and expertise at our institution during the patient’s treatment period, and in later cases, informed patient preference after discussion of both options. This reflects the real-world clinical decision-making process but acknowledges the inherent limitation of selection bias in this retrospective comparison.

### Surgical techniques

#### Uni-portal non-coaxial spinal endoscopic surgery far lateral approach group

The patient was placed in the prone position with the abdomen free. The L5/S1 segment and iliac crest height were localized using C-arm fluoroscopy. General anaesthesia or epidural anaesthesia was administered. Surface landmarks were marked: the posterior midline, the highest point of the iliac crest, the L5 spinous process, and the L5/S1 disc space.

**Incision design:** A longitudinal incision approximately 1.2–1.5 cm long was made approximately 6–8 cm lateral to the posterior midline on the affected side, located in the depression between the L5 transverse process and the sacral ala.

**Surgical steps:** (1) The skin, subcutaneous fascia, and underlying muscles were sequentially incised and dissected bluntly using a soft tissue dissector down to the bone surface. (2) The uni-portal non-coaxial spinal endoscopic system (Arthrex 30° arthroscope) was connected. The endoscope and surgical instruments were introduced through the same working portal, allowing for independent visualization and operation. (3) Anatomical landmarks were identified under endoscopy: the inferior border of the L5 transverse process, the superior border of the sacral ala, the extraforaminal ligaments (lateral intertransverse ligament), and the exit of the L5 nerve root. (4) Soft tissue was cleared using a radiofrequency probe and pituitary forceps to expose the working area between the inferior border of the L5 transverse process and the superior border of the sacral ala. (5) Foraminoplasty was performed using an endoscopic burr or Kerrison punch: a portion of the inferior border of the L5 transverse process, the superior border of the sacral ala, and the hypertrophic tip of the superior articular process were resected to enlarge the external opening of the foramen. (6) The surgical field was explored medially along the course of the L5 nerve root; hypertrophic ligamentum flavum and herniated disc material were resected until the L5 nerve root was fully decompressed, evidenced by visible pulsation. (7) Haemostasis was achieved with radiofrequency ablation, the endoscope was withdrawn, and the incision was sutured.

#### PETD group

**Fig. 1:**
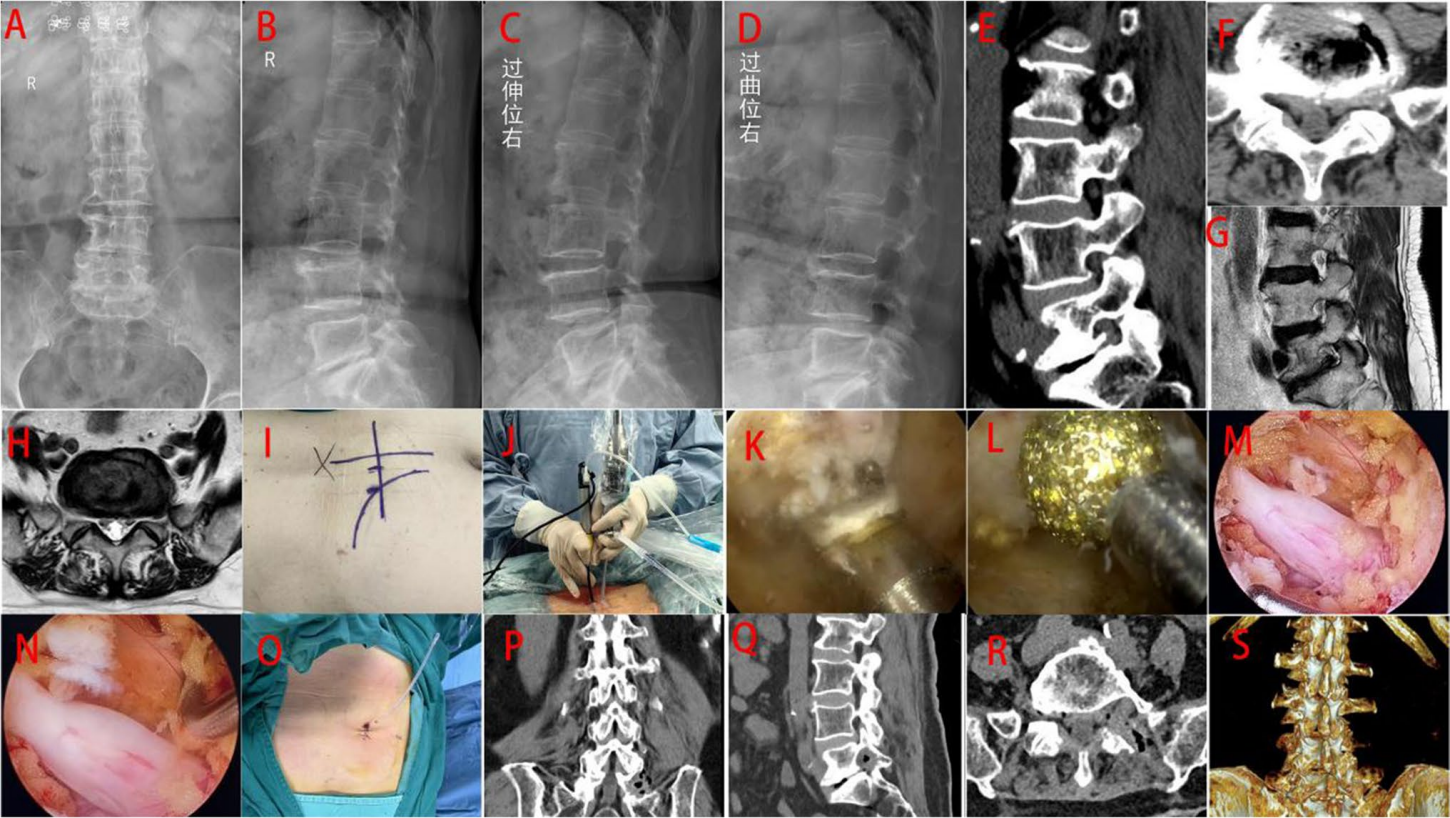
Surgical process of the uni-portal non-coaxial spinal endoscopic surgery far lateral approach group. (**A-H**) Preoperative lumbar X-rays showing a high iliac crest, lumbar degeneration without significant segmental instability; L5/S1 left foraminal stenosis, disc herniation with calcification. (**I**) Intraoperative incision. (**J-L**) Intraoperative procedure. (**M–N**) After nerve root decompression. (**O**) Postoperative incision status. (**P-S**) Findings at the last follow-up

**Fig. 2:**
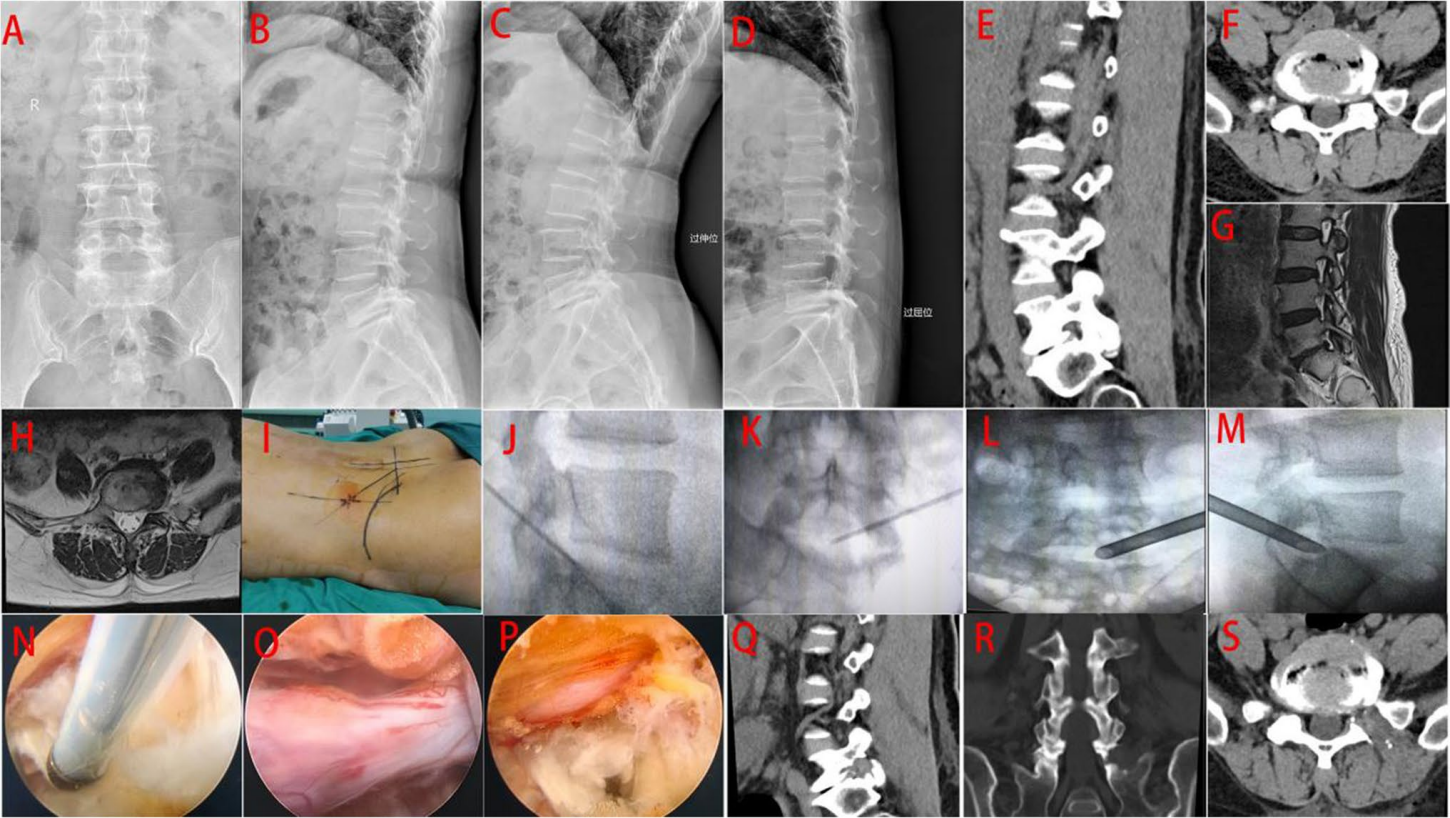
Surgical process of the PETD group. (**A-H**) Preoperative lumbar X-rays showing a high iliac crest, lumbar degeneration without significant segmental instability; L5/S1 left foraminal stenosis. (**I**) Intraoperative incision localization. (**J-M**) Intraoperative puncture and working channel placement. (N-P) Intraoperative nerve root decompression. (**Q-S**) Findings at the last follow-up

The standard transforaminal endoscopic approach (coaxial technique) was performed. Patient positioning was the same as above. Local anaesthesia with sedation or general anaesthesia was used. Under C-arm guidance, the target point for puncture was the Kambin's triangle of the L5/S1 foramen. Due to the high iliac crest, a more cephalad and lateral trajectory was required for puncture. The 45° tilted transforaminal approach was used in some patients, with the puncture point located 8–10 cm lateral to the posterior midline at a 45° angle to the coronal plane to bypass the iliac crest obstruction [22]. Iliac bone drilling or extensive foraminoplasty was performed if necessary. After sequential dilation, a working cannula (approximately 6–7 mm in diameter) was inserted, and the coaxial endoscopic system (joimax) was attached. Foraminoplasty was performed under endoscopy using a reamer or Kerrison punch to expose and decompress the L5 nerve root and dural sac. Herniated disc material was removed. Hemostasis was achieved with radiofrequency ablation, the endoscope was withdrawn, and the incision was sutured (Figs. 1 and 2).

### Perioperative management

Patients were allowed to ambulate 24 h postoperatively and wore a lumbar brace for four weeks. Non-steroidal anti-inflammatory drugs were routinely administered orally for two weeks. Discharge criteria included well-healed incision, no signs of infection, essentially relieved pain, and independent ambulation.

### Outcome measures

#### Perioperative parameters

Operative time (from skin incision to closure),fluoroscopy time (number of C-arm exposures), estimated blood loss (estimated based on suction canister volume and gauze weighing), and length of hospital stay were recorded.

#### Clinical outcome evaluation

Leg pain intensity was assessed using the Visual Analogue Scale (VAS) (0 = no pain, 10 = worst possible pain) preoperatively, and at one week, three months, six months, and 12 months postoperatively. Lumbar functional status was assessed using the Oswestry Disability Index (ODI) (0% = normal function, 100% = completely disabled) preoperatively, and at three months, six months, and 12 months postoperatively. At 12 months postoperatively, the modified MacNab criteria were used to evaluate clinical outcomes: Excellent (complete symptom resolution, return to normal work/life), Good (occasional mild symptoms, no impact on work/life), Fair (symptom improvement but with some functional limitation), Poor (no improvement or worsening of symptoms) [23].

#### Radiological evaluation

All patients underwent lumbar CT and MRI preoperatively, and at three days and 12 months postoperatively. Measured parameters included: (1) Foraminal area: measured by manually tracing the boundaries of the L5/S1 foramen on mid-sagittal images; (2) Facet joint preservation rate: assessed on postoperative CT by measuring the extent of facet joint resection on the surgical side; (3) Segmental stability: measured on dynamic flexion–extension X-rays, assessing translation and angular change at L5/S1. Segmental instability was defined as translation 3 mm or angular change 10°.

#### Complications

Intraoperative complications such as dural tear, nerve injury, vascular injury, and postoperative complications such as paraesthesia, motor weakness, haematoma, infection, recurrence, and reoperation were recorded.

### Statistical analysis

Statistical analysis was performed using SPSS version 26.0. Continuous data were expressed as mean ± standard deviation (mean ± SD). Paired t-tests were used for intragroup comparisons, and independent samples t-tests were used for intergroup comparisons. Categorical data were expressed as frequencies and percentages, and comparisons between groups were performed using the chi-square test or Fisher's exact test. Repeated measures data were analyzed using repeated measures ANOVA. A P-value < 0.05 was considered statistically significant.

## Results

### Comparison of perioperative parameters

All surgeries were completed successfully without conversion to open surgery. The comparison of perioperative parameters is shown in Table 2. The uni-portal non-coaxial spinal endoscopic surgery group had a noticeably shorter operative time (68.5 ± 12.3 min vs. 92.6 ± 18.4 min, P < 0.001) and noticeably fewer intraoperative fluoroscopy (5.4 ± 1.5 vs. 15.8 ± 4.2, P < 0.001) compared to the PETD group. There were no statistically significant differences between the two groups in estimated blood loss (42.5 ± 15.3 mL vs. 40.2 ± 16.1 mL, P 0.05) or length of hospital stay (3.2 ± 1.1 days vs. 3.6 ± 1.4 days, P 0.05).

**Table 2:** Comparison of perioperative parameters between the two groups (mean ± SD)

Parameter	UNSES/AUSS Group (*n* = 42)	PETD Group (*n* = 40)	T value	*P* value
Operative Time (min)	68.5 ± 12.3	92.6 ± 18.4	−6.928	< 0.001
Intraoperative Fluoroscopy (time)	5.4 ± 1.5	15.8 ± 4.2	−14.936	< 0.001
Estimated Blood Loss (mL)	42.5 ± 15.3	40.2 ± 16.1	0.667	0.507
Length of Stay (days)	3.2 ± 1.1	3.6 ± 1.4	−1.449	0.152

The prolonged operative time in the PETD group was mainly attributed to the increased difficulty of puncture due to the high iliac crest, multiple fluoroscopic adjustments of the puncture trajectory, and the time required for extensive foraminoplasty. In contrast, the uni-portal non-coaxial spinal endoscopic surgery group required no complex puncture process as instruments were directly inserted via anatomical landmarks, leading to relatively shorter operative time. Regarding fluoroscopy time, the PETD group required an average of 15.8 fluoroscopic exposures to achieve optimal puncture due to the obstruction of the high iliac crest. Although the 45° tilted transforaminal approach was used in some patients to reduce the fluoroscopy burden, the total number remained noticeably higher than in the uni-portal non-coaxial spinal endoscopic surgery group.

### Comparison of clinical outcomes

Leg pain VAS scores and ODI indices at all postoperative time points were significantly improved compared to preoperative values in both groups (*P* < 0.05). Intergroup comparison showed that at one week postoperatively, the uni-portal non-coaxial spinal endoscopic surgery group had noticeably better leg pain VAS scores than the PETD group (2.2 ± 0.7 vs. 3.3 ± 1.3, *P* < 0.01); at three, six and 12 months postoperatively, there were no significant differences in VAS scores between the two groups (P 0.05) (Table 3). ODI indices showed no significant differences between the two groups at any follow-up time point (P 0.05) (Table 4).

**Table 3:** Comparison of leg pain VAS Scores between the two groups (score, mean ± SD)

Time Point	UNSES/AUSS Group (*n* = 42)	PETD Group (*n* = 40)	T value	*P* value
Preoperative	6.8 ± 1.2	6.9 ± 1.3	−0.363	0.718
1 week postop	2.2 ± 0.7	3.3 ± 1.3	−4.724	< 0.01
3 months postop	1.7 ± 0.6	2.0 ± 0.9	−1.780	0.079
6 months postop	1.4 ± 0.5	1.6 ± 0.7	−1.493	0.140
12 months postop	1.2 ± 0.5	1.4 ± 0.6	−1.667	0.100
F value (time effect)	512.36*	435.82*		
P value	< 0.001	< 0.001		

*Repeated measures ANOVA showed significant differences across time points within each group (*P* < 0.001)

**Table 4:** Comparison of ODI Indices Between the Two Groups (%, mean ± SD)

Time Point	UNSES/AUSS Group (*n* = 42)	PETD Group (*n* = 40)	T value	*P* value
Preoperative	58.5 ± 10.6	59.2 ± 11.2	−0.294	0.770
3 months postop	22.1 ± 6.5	24.5 ± 7.4	−1.585	0.117
6 months postop	16.2 ± 5.3	17.8 ± 6.1	−1.283	0.203
12 months postop	13.8 ± 4.6	15.3 ± 5.4	−1.367	0.176
F value (time effect)	378.45*	335.26*		
P value	< 0.001	< 0.001		

*Repeated measures ANOVA showed significant differences across time points within each group (*P* < 0.001)

At the 12-month follow-up, according to the modified MacNab criteria: In the uni-portal non-coaxial spinal endoscopic surgery group, 28 cases (66.7%) were excellent, ten (23.8%) good, three (7.1%) fair, and one (2.4%) poor, with an excellent-to-good rate of 90.5%. In the PETD group, 23 cases (57.5%) were excellent, 12 (30.0%) good, four (10.0%) fair, and one (2.5%) poor, with an excellent-to-good rate of 87.5%. There was no statistically significant difference in the excellent-to-good rates between the two groups (P 0.05).

### Comparison of radiological outcomes

There was no significant difference in the preoperative L5/S1 foraminal area between the two groups (P 0.05). On CT scans at three days postoperatively, the uni-portal non-coaxial spinal endoscopic surgery group had a noticeably larger foraminal area (79.8 ± 13.2 mm^2^ vs. 63.5 ± 12.1 mm^2^, *P* < 0.001) and a noticeably higher facet joint preservation rate (93.5% vs. 75.8%, *P* < 0.01) compared to the PETD group (Table 5). At the 12-month follow-up, foraminal areas in both groups were slightly reduced compared to three days postoperatively, but the intergroup difference remained statistically significant (*P* < 0.01). Dynamic X-rays showed no cases of segmental instability in the uni-portal non-coaxial spinal endoscopic surgery group, while two cases (5.0%) in the PETD group exhibited segmental instability (translation 3 mm), with no statistically significant difference between the groups (*P* 0.05).

**Table 5:** Comparison of radiological parameters between the two groups (mean ± SD)

Parameter	Time Point	UNSES/AUSS Group (*n* = 42)	PETD Group (*n* = 40)	t value	*P* value
Foraminal Area (mm^2^)	Preoperative	52.3 ± 9.8	53.1 ± 10.2	−0.364	0.717
	3 days postop	79.8 ± 13.2	63.5 ± 12.1	5.893	< 0.001
	12 months postop	77.5 ± 12.3	62.2 ± 11.4	5.924	< 0.01
Facet Preservation Rate (%)	3 days postop	93.5 ± 4.8	75.8 ± 7.6	12.586	< 0.01
Segmental Instability (n, %)	12 months postop	0 (0)	2 (5.0)	-	0.234*

*Fisher's exact test

The lower facet joint preservation rate in the PETD group (75.8%) reflects the necessity for more extensive facet joint resection to establish a working channel under the challenging condition of a high iliac crest. In contrast, the uni-portal non-coaxial spinal endoscopic surgery group, utilizing the far lateral approach, required only minimal resection of the inferior border of the L5 transverse process and the superior border of the sacral ala to gain sufficient operating space, allowing for maximal preservation of the facet joint.

### Complications

Complications occurred in three patients (7.1%) in the uni-portal non-coaxial spinal endoscopic surgery group, including transient postoperative paraesthesia in two cases (4.8%), which resolved with neurotrophic medication within one to three months, and postoperative haematoma in one case (2.4%), which resolved with conservative treatment. Complications occurred in seven patients (17.5%) in the PETD group, including transient postoperative paraesthesia in five cases (12.5%), dural tear in one case (2.5%) that healed with conservative treatment, and postoperative incision infection in one case (2.5%) that healed with dressing changes and antibiotic therapy. There was no statistically significant difference in the overall complication rate between the two groups (P 0.05). There were no cases of permanent neurological injury, discitis, or reoperation.

The higher incidence of transient paresthesia in the PETD group (12.5%) may be related to compression of the exiting L5 nerve root by the working cannula under the high iliac crest condition (Table 6).

**Table 6:** Comparison of complications between the two groups [*n* (%)]

Complication Type	UNSES/AUSS Group (*n* = 42)	PETD Group (*n* = 40)	*P* value
Transient Paresthesia	2 (4.8)	5 (12.5)	0.261
Dural Tear	0 (0)	1 (2.5)	0.488
Postoperative Hematoma	1 (2.4)	0 (0)	1.000
Incision Infection	0 (0)	1 (2.5)	0.488
Total	3 (7.1)	7 (17.5)	0.188

## Discussion

Minimally invasive treatment of L5/S1 foraminal stenosis remains a challenge in spinal surgery. The anatomy of this segment is complex, with a high iliac crest, a steep course of the L5 nerve root, and a narrow foramen. The traditional transforaminal approach often encounters difficulty in optimal working cannula placement due to iliac bone obstruction [24, 25]. This study compared the clinical outcomes of the far lateral approach of uni-portal non-coaxial spinal endoscopic surgery and PETD for treating L5/S1 foraminal stenosis with a high iliac crest. The results show that both techniques can achieve satisfactory outcomes, but uni-portal non-coaxial spinal endoscopic surgery offers notable advantages in terms of surgical efficiency, fluoroscopic burden, completeness of decompression, and protection of the facet joint.

### Impact of high iliac crest on the L5/S1 transforaminal approach

A high iliac crest is the most common anatomical obstacle for endoscopic surgery via the transforaminal approach at L5/S1. According to the literature, approximately 15%−30% of the Asian population has a high iliac crest, a rate significantly higher than in Western populations [26, 27]. A high iliac crest necessitates excessive cephalad and lateral angulation of the puncture needle and working cannula to enter the foramen. This not only increases the difficulty of puncture and fluoroscopy but can also lead to compression of the exiting L5 nerve root by the working cannula, resulting in postoperative paraesthesia or motor deficits [28]. To overcome this obstacle, surgeons often must perform extensive foraminoplasty or even partial resection of the superior articular process, which increases the risk of postoperative segmental instability [29].

Various modified puncture techniques have been proposed to address the high iliac crest issue. Among them, the 45° tilted transforaminal approach (45°TELD), with the puncture point located 8–10 cm lateral to the posterior midline at a 45° angle to the coronal plane, effectively avoids iliac crest obstruction, shortens the puncture path, and reduces fluoroscopy. The Gu percutaneous endoscopic technique involves dynamic adjustment of the puncture direction combined with a "down-pressing foraminoplasty" technique, suitable for complex cases where anatomical landmarks are unclear due to obesity or severe degeneration.

In this study, the PETD group required a high number of intraoperative numbers of fluoroscopy (15.8 ± 4.2), notably more than the uni-portal non-coaxial spinal endoscopic surgery group, reflecting the difficulty of puncture under the high iliac crest condition. Even with the 45° tilted approach, multiple fluoroscopic confirmations were often needed due to individual anatomical variations. Furthermore, the facet joint preservation rate in the PETD group was only 75.8%, lower than the 93.5% in the uni-portal non-coaxial spinal endoscopic surgery group, suggesting that PETD causes elevated destruction of the facet joint during decompression. This is consistent with the findings of Yang et al. [30], who reported that even with visualization using a reamer, the extent of facet joint resection during endoscopic decompression at L5/S1 was moderatedly elevated in patients with a high iliac crest compared to those without.

### Anatomical basis and technical advantages of the far lateral approach of uni-portal non-coaxial spinal endoscopic surgery

The design of the far lateral approach of uni-portal non-coaxial spinal endoscopic surgery is based on a thorough understanding of the anatomy surrounding the lumbar spine. This approach utilizes the Wiltse muscle-splitting interval (between the multifidus and longissimus muscles) to directly reach the intertransverse region, with the working portal located lateral to the foramen, completely bypassing the iliac crest [31]. Clear anatomical landmarks are visible under endoscopy: the inferior border of the L5 transverse process, the superior border of the sacral ala, the lateral intertransverse ligament, and the exit of the L5 nerve root form a stable working zone.

Unlike traditional coaxial endoscopy (e.g., PETD), uni-portal non-coaxial spinal endoscopic surgery employs a non-coaxial design with distinct technical features: (1) Separation of visualization and operation: the endoscope and instruments enter through the same working portal but function independently; the endoscope can be moved independently, providing multi-angle visualization, while instruments have greater freedom of movement; (2) The larger-diameter working channel through the muscle allows for the use of various instruments, improving surgical efficiency; (3) High irrigation fluid flow ensures a clear visual field, facilitating precise anatomical dissection; (4) The haptic feedback is similar to that of open surgery, resulting in a relatively gentle learning curve; (5) Combining the visual advantages of biportal endoscopy with the minimally invasive nature of uniportal endoscopy, it is particularly suitable for revision surgeries and complex reconstructions (e.g., annulus fibrosus repair, dural repair).

The technical advantages of this approach include: (1) Unrestricted by iliac crest height, making it suitable for various iliac crest morphologies; (2) The working channel directly targets the extraforaminal opening, allowing "retrograde" decompression along the course of the L5 nerve root from outside inwards. The decompression direction aligns with the nerve root trajectory, enhancing safety; (3) The entire course of the L5 nerve root, including the exit, traversing, and entry zones, can be clearly visualized under endoscopy, enabling true full-foraminal decompression [32]; (4) By resecting a portion of the inferior border of the L5 transverse process and the superior border of the sacral ala to expand the working space, extensive facet joint resection is avoided, better preserving the integrity of the posterior column structures.

The radiological results of this study showed a noticeably larger postoperative foraminal area in the uni-portal non-coaxial spinal endoscopic surgery group compared to the PETD group (79.8 ± 13.2 mm^2^ vs. 63.5 ± 12.1 mm^2^, *P* < 0.001), indicating that uni-portal non-coaxial spinal endoscopic surgery achieves more thorough foraminal decompression. This is consistent with the anatomical study by Kim et al. [33], which concluded that the far lateral approach provides a wider exposure of the L5/S1 foramen compared to the transforaminal approach, especially for decompression of the extraforaminal zone.

### Analysis of clinical outcomes

Both groups showed significant improvement in leg pain VAS scores and ODI indices postoperatively compared to preoperatively, with excellent-to-good rates at 12 months of 90.5% and 87.5%, respectively. This indicates that both techniques are effective for treating L5/S1 foraminal stenosis with a high iliac crest. Intergroup comparison showed that the uni-portal non-coaxial spinal endoscopic surgery group had more favourable leg pain VAS scores at one week postoperatively (2.2 ± 0.7 vs. 3.3 ± 1.3, *P* < 0.01), possibly due to less intraoperative nerve root manipulation and milder early postoperative inflammatory response. As follow-up time increased, the clinical outcomes between the two groups converged, suggesting that although PETD is technically more challenging intraoperatively, it can ultimately achieve decompression results comparable to uni-portal non-coaxial spinal endoscopic surgery.

Notably, the incidence of transient postoperative paraesthesia was higher in the PETD group (12.5%) than in the uni-portal non-coaxial spinal endoscopic surgery group (4.8%), although the difference did not reach statistical significance. This still reflects the clinical reality of elevated disturbance to the exiting nerve root with the transforaminal approach under high iliac crest conditions. A large case series by Lee et al. [34] also reported that the incidence of postoperative paresthesia after transforaminal approach at L5/S1 could be as high as 8%−15%, significantly higher than at other levels.

Although the UNSES/AUSS group achieved a notably larger postoperative foraminal area and higher facet joint preservation rate, the long-term clinical outcomes (ODI, MacNab criteria) at 12 months were comparable between the two groups. This suggests that once adequate neural decompression is achieved, additional enlargement of the foramen may not translate into measurable extra clinical benefit in terms of patient-reported function. The clinical significance of the radiological differences therefore requires careful interpretation. However, better facet joint preservation could theoretically confer a long-term biomechanical advantage by reducing the risk of future segmental instability, a hypothesis that requires validation through studies with longer follow-up.

### Technical comparison between uni-portal non-coaxial and coaxial endoscopy

Uni-portal non-coaxial spinal endoscopic surgery, as a representative of uniportal non-coaxial spinal endoscopy, differs from traditional coaxial endoscopy (PETD) in several aspects:

1. **Optical System:** Coaxial endoscopy has the lens and working channel aligned, with the visual field center aligned with the instrument direction, but the viewing angle is limited. Non-coaxial endoscopy allows for independent movement of the lens, providing a wider viewing angle and better visualization.
2. **Operating Space:** Coaxial endoscopy uses a relatively small working channel (typically 4–7 mm), limiting instrument maneuverability. Non-coaxial endoscopy utilizes a larger working channel through muscle (up to 8–15 mm), allowing the use of instruments similar to those in open surgery, offering greater operational flexibility.
3. **Indications:** Coaxial endoscopy is better suited for simple disc herniation and intraforaminal lesions. Due to its larger working space, non-coaxial endoscopy has broader indications, including foraminal stenosis, facet hypertrophy, far-lateral disc herniation, revision surgeries, annulus fibrosus repair, and dural repair.
4. **Learning Curve:** Coaxial endoscopy requires mastering specific eye-hand coordination, leading to a steeper learning curve. The haptic feedback and operational feel of non-coaxial endoscopy are closer to microscopic surgery, making it easier for beginners to learn.
5. **Fluoroscopy Dependence:** Coaxial endoscopy is highly dependent on intraoperative fluoroscopic localization, especially in patients with a high iliac crest, significantly increasing the number of exposures. Non-coaxial endoscopy relies on anatomical landmarks for localization, requiring less fluoroscopy.
6. **Facet Joint Preservation:** Coaxial endoscopy often requires partial resection of the facet joint to establish the working channel. Non-coaxial endoscopy, utilizing the intertransverse approach, preserves the facet joint more completely.

In this study, the uni-portal non-coaxial spinal endoscopic surgery group had a noticeably shorter operative time than the PETD group (68.5 ± 12.3 min vs. 92.6 ± 18.4 min, P < 0.001), reflecting the operational efficiency advantage of the non-coaxial design. The lower fluoroscopy time in the uni-portal non-coaxial spinal endoscopic surgery group is due to the fact that the approach does not require complex puncture localization; the working channel can be established directly via anatomical landmarks.

The shorter operative time and reduced fluoroscopy with the UNSES/AUSS approach must be interpreted in the context of the surgical learning curve. PETD for L5/S1 with a high iliac crest is widely recognized as one of the most challenging endoscopic procedures, often requiring advanced foraminoplasty skills. In contrast, the far lateral approach utilizes a more familiar anatomical corridor (Wiltse interval) for many spine surgeons. Thus, the observed efficiency may be attributable to both the intrinsic advantages of the approach and a relatively less steep learning curve for surgeons already skilled in open or microscopic paraspinal techniques. The choice of technique should, therefore, consider surgeon experience and comfort alongside patient anatomy.

### Indications and technical limitations

Based on our experience and the literature, the main indications for uni-portal non-coaxial spinal endoscopic surgery include: (1) L5/S1 foraminal stenosis with a high iliac crest; (2) Far-lateral disc herniation; (3) L5 nerve root exit zone entrapment (far-out syndrome); (4) Revision surgery after posterior approaches requiring avoidance of scar tissue; (5) Cases requiring complex reconstruction (e.g., annulus fibrosus repair, dural repair).

Relative limitations of this technique include: (1) Limited ability to decompress the central canal and lateral recess; if significant central stenosis is present, it may require combination with other approaches; (2) The working area is adjacent to the L5 nerve root and segmental artery, requiring precise manipulation to avoid injury; (3) The endoscopic viewing direction differs from the traditional transforaminal approach, requiring adaptation for beginners.

### Study limitations and future directions

This study has several limitations that must be considered when interpreting the results. First and foremost, its retrospective and non-randomized design introduces the potential for selection bias. The choice of surgical technique (UNSES/AUSS vs. PETD) was likely influenced by surgeon preference, evolving institutional expertise, and specific patient anatomical factors not fully captured in our inclusion criteria. This limits the strength of causal inferences regarding the superiority of one technique over the other. Second, the sample size, while adequate for detecting large differences in perioperative parameters, may be underpowered to reveal more subtle differences in long-term clinical outcomes or rare complications. Third, the follow-up duration of 12 months, while sufficient to assess early and mid-term efficacy, is too short to evaluate long-term sequelae such as segmental instability, adjacent segment disease, or late recurrence. Fourth, all procedures were performed by surgeons experienced in endoscopic spine surgery. The significant difference in operative time may partly reflect a learning curve effect, as the PETD procedure is notoriously more challenging at L5/S1 with a high iliac crest. The efficiency of the UNSES/AUSS approach in our series might not be generalizable to centers beginning their experience with this technique. Finally, we did not perform a formal cost-effectiveness analysis, which is an important consideration for healthcare systems. Future prospective, randomized controlled trials with larger cohorts, longer follow-up, and multi-centre designs are warranted to validate our findings and more definitively establish the comparative effectiveness and indications for each technique.

## Conclusion

Both the far lateral approach of uni-portal non-coaxial spinal endoscopic surgery and PETD are effective treatments for L5/S1 foraminal stenosis with a high iliac crest, yielding satisfactory clinical outcomes. Both techniques yielded satisfactory and comparable long-term clinical outcomes. However, the UNSES/AUSS far lateral approach demonstrated distinct perioperative advantages, including shorter operative time, significantly less fluoroscopy exposure, more extensive foraminal decompression radiologically, and better preservation of the facet joint. These features may render it a particularly valuable and efficient option for surgeons proficient in this technique when treating L5/S1 foraminal stenosis with a high iliac crest, especially in patients where minimizing radiation and preserving posterior structures are prioritized.

## Data Availability

No datasets were generated or analysed during the current study.
